# Supplementary material for: Food taboos during pregnancy: meta-analysis on cross cultural differences suggests specific, diet-related pressures on childbirth among agriculturalists
Source: PeerJ. 2022 Jul 11;10:e13633. doi: 10.7717/peerj.13633 (PMC9281602; doi:10.7717/peerj.13633)
Supplement: Supplemental Information 3 [file peerj-10-13633-s003.docx]

1. The rationale for conducting the systematic review / meta-analysis;

The tendency of many societies to solicit a restriction of maternal dietary intake during pregnancy through cultural norms, namely food taboos, is witnessed by several articles in the existing literature. While the literature on origin and purposes of general food taboos is rich even at a comparative level [(e.g. Navarrete & Fessler, 2003; Simoons, 1994; (Spielmann, 1989)](https://paperpile.com/c/2qmpvk/fI6S+Bxk6), a preliminary cross cultural comparison of similarities and differences in avoided foods during pregnancy and reasons for the avoidance is limited to two studies (Carles, 2014; Iradukunda, 2019). The fundamental role of maternal dietary behaviour on pregnancy outcome, maternal-infant life history, and fitness should rather increase the academic interest in understanding which foods are avoided during pregnancy and why across the world. While all taboos can be shaped by both social and biological interests, we assume that those during pregnancy are more likely to reveal perceived biological stresses during pregnancy across human populations, due to the specific biological importance of pregnancy in relation to other less sensitive life-stage targeted by food restrictions. The reviews available do not provide systematic approach and statistic methods to detect which foods are perceived as dangerous during pregnancy and why. Therefore, we propose the first meta-analysis on currently available literature to shade light on perceived biological pressures on pregnancy across the world and discuss implications for future research in evolutionary and global health issues.

1. The contribution that it makes to knowledge in light of previously published related reports, including other meta-analyses and systematic reviews.

Current works on cross cultural food taboos during pregnancy (Carles, 2014; Iradukunda, 2019) provide a qualitative discussion on most common avoided foods and reasons, but they do now provide the following statistical information: frequency of taboo food types avoided around the world; a reduction of current information on reasons for taboos into focus categories in order to detect trends in perceived dangers, to be investigated in general and in relation to each avoided food type; a comparison of food taboos and reasons among subsistence modes (agriculture and foraging) to assess whether different strategies of food supply can affect food taboos during pregnancy. We contributed to fill the gap in meta-analysis on dietary restrictions during pregnancy by reviewing 32 articles that mentioned food taboos during pregnancy together with reasons for the ban. Despite current literature on dietary behaviour is sparse, our approach was useful to: (i) illustrate the first quantitative distribution of avoided animal, plant and miscellaneous products; (ii) highlight that reasons for food avoidance follow a first time identified qualitative gradient in terms of specificness and likeness of physiological dangers; (iii) provide a first quantitative evidence that the avoidance of carbohydrate rich food to avoid difficult delivery is a cross cultural trend that is more consistent and specific than other antenatal food restrictions, and this confirms the need for major attention on reduced dietary intake during pregnancy and obstructed labour in low income countries as advanced by Dove (2010) and Rush (2000); (iv) provide first evidence that subsistence modes can shape culturally transmitted behaviour during pregnancy and that the major avoidance of carbohydrate during pregnancy to avoid increased infant birth weight among agriculturalists overlaps both the clinical evidence that carbohydrates contribute to obstructed labor and the evolutionary perspective that obstructed labor was exacerbated by agriculture and industrialization. Our main contribution is thus to provide a first systematic and statistically defined overlook at the distribution of food taboos during pregnancy, which lead to (i) discuss examples of their biological implications, (ii) highlight the issue of obstructed labor as point of convergence of global health, evolutionary aspects, and phenotypic plasticity. Our study paves the way for future investigation on determinants of changes in dietary behaviour during pregnancy and obstructed labor at population and comparative level.
